# Paediatric Practitioners' Acceptance of the National Paediatric Early Warning System (PEWS)

**DOI:** 10.1111/nicc.70547

**Published:** 2026-06-16

**Authors:** Linda K. Kaye, Lyvonne N. Tume, Peter White

**Affiliations:** ^1^ Department of Psychology Edge Hill University Lancashire UK; ^2^ School of Nursing and Midwifery Education Edge Hill University Lancashire UK; ^3^ Paediatric Intensive Care Unit Alder Hey Children's Hospital Liverpool UK; ^4^ Digital and Innovation Team Alder Hey Children's Hospital Liverpool UK

**Keywords:** digital acceptance, paediatric care, PEWS, psychology, technology acceptance

## Abstract

Early detection of deterioration in children in hospital is essential for improving patient outcomes, prompting NHS England to introduce a digital National Paediatric Early Warning System (PEWS) in November 2023. Our service evaluation examined a locally delivered digital version of the PEWS system, introduced in July 2025 to explore paediatric staffs' acceptance before and after implementation, measured by the Technology Integration Evaluation Resource (TIER), based on the Technology Integration Model. Online surveys were completed pre‐ and post‐implementation. Acceptance did not significantly change over time. However, user motivation, agency and perceived cost–benefit as well as perceptions of PEWS as an extension of self, were positively associated with acceptance, highlighting key psychological factors influencing digital health adoption.

## Impact Statement

- What is Known About This Topic?
  ○ A human factors approach to PEWS adoption can increase system utilisation and return on investment.
- What this paper adds?
  ○ Understanding psychological barriers to digital health adoption can identify more targeted implementation strategies both at an individual and organisational level.

## 1 Background and Rationale

Early detection of physiological deterioration in hospitalised children is crucial in ensuring timely critical care unit admission and optimising outcomes [1, 2, 3]. Although our hospital had used a PEWS in both paper and electronic form since 2006, it was believed that standardisation of PEWS used across England was needed. Thus, in 2025, NHS England implemented a national standardised approach [4]; the digital National Paediatric Early Warning System (PEWS).

However, as is the case for any type of digital implementation, it is vital to understand users' *acceptance* of these. Increasing research has examined technology acceptance [5, 6, 7, 8, 9] and the psychological factors relating to technology adoption. The Technology Integration Model (TIM; [10]) is such a model which has been applied to understanding acceptance of digital health systems [11]. Our project applied the TIM to understand paediatric staffs' acceptance of PEWS. This evaluation was situated to measure acceptance both before and after the initiation of a new digital method of PEWS delivery. We asked:

1. What are the key barriers and enablers associated with PEWS?
2. To what extent are there changes in acceptance of PEWS over time?

## 2 Method and Analysis

### 2.1 Study Design and Sample

Prior to commencement, our project was registered as a service evaluation with Alder Hey Children's Hospital (Service Evaluation ID reference number: 7364). This evaluation is reported according to the SQUIRE reporting checklist.

Paediatric clinical staff (*N* = ~1400) who represented the following sub‐groups were invited to take part in an online survey: paediatric nurses (staff nurse to nurse manager level), non‐qualified nursing support staff student nurses, and the staff who respond to PEWS triggers, comprising medical staff, advanced nurse practitioners and the hospital Medical Emergency/Outreach team (who intercept warning alerts). The survey was completed at two time points; May 2025 (T1), prior to new delivery roll‐out, and from August 2025 (T2) at post‐roll‐out. At each time point, the survey took approximately 10 min to complete. A total of 124 participants took part in the survey over the two time points (*n* = 62 at both T1 and T2), but after removing incomplete data, this resulted in a final sample of 55 in total.

The 21‐item survey is comprised of a short demographics section and the Technology Integration Evaluation Resource (TIER) to measure users' perceptions of factors associated with acceptance of PEWS, which is based on factors of the TIM [10]. See Appendix 1 for TIER items. This includes the following factors: technological features and functions, user agency, situational context, user motivation, cost–benefit analysis (extent of deliberate decision‐making) and self‐extension (extent to which technology system extends vs. reduces capabilities). Mean scores were calculated to generate a total acceptance score (TIER total) and for each sub‐scale. The TIER scale showed good levels of internal consistency, with a Cronbach's α of 0.73 (Time 1) and 0.74 (Time 2).

### 2.2 Data Analysis

To evaluate any change in acceptance between Time 1 and Time 2, a MANOVA was conducted. Additionally, to understand what were perceived key barriers and enablers of acceptance, we used the median for each of the TIER scales to identify frequencies of responders who scored above versus below this at each time‐point. TIER variables with a significantly higher frequency of high compared to low responses is indicative of the TIER variable being an enabler. To assess any differences in frequencies of high vs. low responses between Time 1 and Time 2, a Chi‐squared test was performed for total TIER as well as the sub‐scales. Finally, to assess the extent to which high and low scores (determined by median split) on each TIER sub‐scale related to overall PEWS acceptance, we conducted a Pearson's correlation.

## 3 Results

At Time 1 (*N* = 34), the majority of participants were female (97.1%), with an average age of 37.53 years (SD = 10.23), and an average length of service in their respective role at the trust of 6.87 years (SD = 7.83). In terms of role at the trust, the majority of respondents were nurses (*n* = 27), most of whom were staff nurses (*n* = 17) with the remaining participants being care assistants (*n* = 3) and response team members (*n* = 4). At Time 2 (*N* = 21), all participants were female, with an average age of 35.05 years (SD = 9.90), and an average length of service in their respective role at the trust of 7.74 years (SD = 6.97). In terms of role at the trust, the majority of respondents were nurses (*n* = 18), most of whom were staff nurses (*n* = 11) with the remaining participants being care assistants (*n* = 3).

### 3.1 Change in Acceptance

Change analysis revealed that there were no significant differences in PEWS acceptance between Time 1 and Time 2 *F* (6, 36) = 1.22, *p* = 0.317; Wilk's Λ = 0.83, partial *η* ^2^ = 0.169 (Table 1).

**TABLE 1:** Descriptive analysis of acceptance factors.

Scale/sub‐scale	Time 1			Time 2			Mean change
	*M*	SD	Median	*M*	SD	Median	
Total TIER	4.23	0.58	4.33	3.96	0.71	4.12	−
Technological features	5.85	1.36	6.00	5.76	1.22	6.00	−
Agency	4.77	0.83	4.50	4.29	1.04	4.00	−
Extension of self	4.26	1.74	4.40	3.58	1.50	4.00	−
Situational context	3.73	0.91	3.88	3.87	1.09	3.75	+
Motivation	2.68	1.09	2.50	2.69	1.02	3.00	+
Cost–benefit analysis	4.50	1.50	4.50	3.57	1.50	4.00	−

### 3.2 Key Barriers and Enablers

When using the median values to determine the frequency of cases that were enablers (greater frequency of high scores) versus barriers (greater frequency of low scores) for acceptance, enablers were agency (T1), extension of self (T1&T2), motivation (T2) and cost–benefit analysis (T2). Barriers were motivation (T1), technological features (T1&T2), agency (T2) and situation context (T2). However, our inferential analysis did not find any significant changes in the proportion of high versus low scores per variable between time points 1 and 2^1^ (see Table 2).

**TABLE 2:** Frequencies of responders for high versus low per TIER factor between time points.

Scale/sub‐scale	Time 1			Time 2		
	Median	High (*n*)	Low (*n*)	Median	High (*n*)	Low (*n*)
Total TIER	4.33	12	10	4.12	11	10
Technological features	6.00	14	20	6.00	9	12
Agency	4.50	17	12	4.00	9	12
Extension of self	4.40	12	10	4.00	13	8
Situational context	3.88	11	11	3.75	10	11
Motivation	2.50	9	13	3.00	11	10
Cost–benefit analysis	4.50	11	11	4.00	13	8

### 3.3 Factors on Digital Acceptance

At Time 1, we found that extension of self (*r* = 0.59, *p* < 0.01), and motivation (*r* = 0.57, *p* < 0.01) were positively related to acceptance. At Time 2, similarly, we found extension of self (*r* = 0.55, *p* < 0.05) and motivation (*r* = 0.75, *p* < 0.001) to be positively related to PEWS acceptance, in addition to agency (*r* = 0.56, *p* < 0.01) and cost–benefit analysis (*r* = 0.68, *p* < 0.001).

## 4 Discussion

Overall, we did not find that paediatric staff's acceptance changed significantly from pre‐ to post‐roll‐out of the new national PEWS system. This might be explained by the fact that acceptance at Time 1 was relatively high for most TIER factors (with most items averaging above the mid‐point of the scale), indicating that the baseline acceptance was perhaps already established to some extent or other. Interestingly, practitioners' perceptions of PEWS being an extension of self, and its relationship to overall acceptance demonstrates that there are important identity aspects to consider in the use of digital systems when used in organisational contexts. Additionally, perceptions of agency and being in control of PEWS were found to be important post‐roll‐out, indicating that users' self‐efficacy of using digital systems is also a key factor towards overall acceptance.

## 5 Limitations

This was only a small‐scale evaluation, conducted within a single institution with a small sample size, which may limit the generalisability of the findings to other settings.

## 6 Conclusion

Overall, we note the value of adopting a psychologically informed perspective to understand factors that might support digital transformation within critical care settings. This suggests the need to focus not only on the technical aspects of digital system implementation but also the human factors relating to practitioners' self‐efficacy, identity and motivation relating to its use in the paediatric care context.

## Funding

The authors have nothing to report.

## Ethics Statement

As this is a change in the existing service at Alder Hey Children's Hospital, the project was registered as a service evaluation with the trust. This was reviewed and approved by the Clinical Audit Team within the Trust's Governance and Quality Assurance Department (Service Evaluation ID reference number: 7364).

## Consent

The authors have nothing to report.

## Conflicts of Interest

The authors declare no conflicts of interest.

## Data Availability

The data that support the findings of this study are available from the corresponding author upon reasonable request.

## Endnotes

^1^
Specific values are: total TIER: *χ* ^*2*^ (1), *N* (43), =0.020, *p* = 0.887; technology features: *χ* ^*2*^ (1), *N* (55), = 0.015, *p* = 0.920; agency: *χ* ^*2*^ (1), *N* (50), = 1.21, *p* = 0.271; extension of self: *χ* ^*2*^ (1), *N* (43), =0.239, *p* = 0.625; situational context: *χ* ^*2*^ (1), *N* (43), = 0.024, *p* = 0.876; motivation: *χ* ^*2*^ (1), *N* (43), = 0.720, *p* = 0.396; cost–benefit analysis: *χ* ^*2*^ (1), *N* (43), =0.617, *p* = 0.432.

## Appendix 1 TIER Scale

The following questions are asking about your perceptions of using the vitals system (PEWS). Please consider each of the following statements and indicate the extent to which you agree with them, using the following scale: 1 = strongly disagree, 7 = strongly agree.

I feel I can effectively use the features and functions of vitals.

I find that the features and functions of vitals are intuitive to use and easy to navigate.

When using vitals, I feel it is out of my control (R).

I can make vitals work for me rather than the other way around.

The physical space I am in affects how I use vitals.

The cultural, social and/or economic context affects how I use vitals.

The organisational culture influences how I use vitals.

The day or time of day influences how I use vitals.

I use vitals because I need to undertake work tasks.

I use vitals because I experience enjoyment and satisfaction from doing so.

Using vitals benefits me because the features and functions satisfy my reasons for using them.

Please consider the following graphic that includes a set of figures with two circles which vary in the degree of overlap between them. The white circle represents YOU (yourself), and the dark circle represents vitals. When the circles are totally separate, this represents yourself being completely independent from vitals. If the circles are totally overlapping, this represents yourself being completely identified with vitals. Each of the figures is numbered below. Please respond to the following items using the number of the figure which best represents the degree to which you feel there is overlap between yourself and vitals.

Please respond to the following items using the number of the figure which best represents the degree to which you feel there is overlap between yourself and the vitals system (PEWS). Please use the visual above to make a judgement and select the corresponding number as your response.

How closely do vitals correspond with your identity as a paediatric practitioner?

How closely do vitals correspond with your thoughts as a paediatric practitioner?

How closely do vitals correspond with your physical body as a paediatric practitioner?

How closely do vitals correspond to being part of your possessions as a paediatric practitioner?

How closely do vitals correspond to part of your environment as a paediatric practitioner?
